# Drug resistance of *Mycoplasma genitalium*: mechanism and regional distribution

**DOI:** 10.3389/fcimb.2026.1789671

**Published:** 2026-08-25

**Authors:** Yuhang Ouyang, Yujin Zhang, Zhixian Long, Xuan Wu, Ningyuan Li, Xulin Liu, Jin Lin, Yanan Niu, Jiaxin Chen, Jiaxuan Tang, Chunlan Xiao, Peng Liu, Yaliu Zhang, Weidong Gong

**Affiliations:** 1 Hunan Provincial Key Laboratory for Special Pathogens Prevention and Control and Institute of Pathogenic Biology, Basic Medical School, Hengyang Medical School, University of South China, Hengyang, Hunan, China; 2 Department of Thyroid and Breast Surgery, Affiliated Hengyang Hospital of Hunan Normal University & Hengyang Central Hospital, Hengyang, Hunan, China; 3 Department of Clinical Laboratory Medicine, Institution of Microbiology and Infectious Diseases, The First Affiliated Hospital, Hengyang Medical School, University of South China, Hengyang, Hunan, China

**Keywords:** antibiotic resistance, fluoroquinolones, macrolides, *Mycoplasma genitalium*, regional distribution, resistance mechanisms

## Abstract

*Mycoplasma genitalium* is a significant sexually transmitted pathogen associated with various urogenital diseases and severe complications in both men and women. In recent years, advances in molecular diagnostic technologies have progressively revealed its global prevalence. Concurrently, the escalating issue of antibiotic resistance poses a major public health challenge. This review aims to summarize the clinical manifestations and resistance mechanisms of *M. genitalium* infection, the global epidemiological characteristics of antibiotic resistance, as well as the limitations and challenges of existing research, thereby providing a scientific basis for formulating effective control measures. Key acquired resistance mechanisms of *M. genitalium* include macrolide resistance due to 23S rRNA gene mutations; fluoroquinolone resistance triggered by *parC* and *gyrA* gene mutations; and tetracycline resistance associated with the mutations in the 16S rRNA gene. Major external drivers of resistance emergence and spread include antibiotic misuse, sexual transmission networks, latent transmission by asymptomatic carriers, and limitations in detection methods. In recent years, *M. genitalium* infections have been prevalent in many countries worldwide, with rising resistance rates observed in some regions. Resistance rates remain persistently high in Oceania, Asia, and parts of North America, while regions such as Africa exhibit relatively lower levels of resistance. The clinical management of *M. genitalium* infections exhibits significant heterogeneity across major countries in North America, Europe, and the Asia-Pacific region. These differences primarily stem from regional variations in antibiotic resistance patterns, drug accessibility, and differing approaches to antimicrobial stewardship. Antibiotic resistance in *M. genitalium* has become an urgent global public health issue. Future efforts should prioritize strengthening global collaboration on antimicrobial resistance surveillance, promoting the application of molecular epidemiological methods in future research, deepening research into resistance mechanisms, advancing rapid diagnostic technologies and novel drug development, and refining regional clinical management guidelines based on local resistance patterns to effectively curb this evolving threat.

## 1 Introduction

*Mycoplasmas* belong to the class Mollicutes (Chen et al., 2025a), which together with other wall-less bacteria such as *Spiroplasma* (Liu et al., 2022; You et al., 2024) and *Phytoplasma* (Pedroncelli et al., 2026), are characterized by the small size of their genomes and are widely distributed in human and animal hosts (Barré et al., 2004). In humans, five pathogenic *Mycoplasma* species (*Mycoplasma genitalium* (Chen et al., 2025b), *Mycoplasma hominis* (Qiu et al., 2024), *Mycoplasma penetrans* (Li et al., 2025; Zhang et al., 2025) and *Ureaplasma urealyticum* (Dai et al., 2015)) are associated with respiratory tract and urinary and reproductive tracts diseases characterized by high morbidity and low mortality (Wang et al., 2023; Yang et al., 2024); infections can be systemic in neonates and immunocompromised patients. *M. genitalium* was initially isolated and identified in male patients with non-gonococcal urethritis (NGU) in 1981 (Fernández-Huerta et al., 2025). Extensive research has confirmed that *M. genitalium* is a significant pathogen in acute and chronic NGU in men. Increasing evidence also indicates that it can cause cervicitis in women (Yu et al., 2023). It is also significantly associated with serious complications such as pelvic inflammatory disease (PID), tubal infertility, adverse pregnancy outcomes (De Carvalho et al., 2020; Yu et al., 2023) and increased risk of HIV transmission (Zhao et al., 2019; De Carvalho et al., 2020; Boujemaa et al., 2023). Due to the characteristics of *M. genitalium*, including persistent infection, spontaneous clearance, and a high rate of asymptomatic infection, the natural course of untreated infection remains poorly understood (Gnanadurai and Fifer, 2020). With advances in molecular diagnostic technologies, particularly the development and application of polymerase chain reaction (PCR) and nucleic acid amplification testing (NAAT), the true natural history and prevalence of *M. genitalium* infection are increasingly being revealed.

However, because *M. genitalium* lacks a cell wall, it is naturally ineffective against β-lactam antibiotics and other substances that act on the cell wall, greatly limiting the range of treatment options. Extensive data indicate that this pathogen has become the second most common bacterial sexually transmitted infections (STIs) globally after *Chlamydia trachomatis*, with infection rates in the general population typically exceeding those of *Neisseria gonorrhoeae*, constituting a significant public health burden that cannot be ignored (Manhart, 2013; Unemo et al., 2017; Ring et al., 2022; Bébéar et al., 2023). Macrolides, fluoroquinolones and other drugs form the cornerstone of the first-line and second-line treatments recommended by current international guidelines (Drew and Eogan, 2024). Over the past two decades, the emergence of antibiotic resistance to *M. genitalium* has raised concerns about the long-term efficacy of current treatment strategies (Rudman et al., 2025). Resistance to these key antibiotic categories is showing a severe trend of sharp increase and widespread worldwide, with declining cure rates for first-line antimicrobials (Abavisani and Keikha, 2023). This phenomenon is partly attributable to the widespread use of single-dose azithromycin in sexual health settings. It is worth noting that a systematic review and meta-analysis indicated that the antibiotic resistance of *M. genitalium* is not uniformly distributed but shows significant regional differences (Machalek et al., 2020). Another systematic review and meta-analysis published in Lancet Microbe revealed that macrolide resistance rates have increased over time in Europe. Fluoroquinolone resistance rates are highest in the Western Pacific region and show an upward trend in non-Nordic Europe (Chua et al., 2025b). Therefore, clarifying the regional distribution patterns of *M. genitalium* resistance, investigating the diversity of its resistance mechanisms, and comparing differences in treatment guidelines across multiple countries and regions are crucial for developing precise, effective, and locally tailored infection control strategies.

In this review we summarize relevant articles published in the PubMed, Medline and Embase databases. The search combined Medical Subject Headings (MeSH) terms and free-text terms, including “*Mycoplasma genitalium*”, “antimicrobial resistance”, and “geographic location” (such as “country” and “prevalence by region”). To facilitate the analysis of regional differences, priority was given to studies reporting resistance rates stratified by geographical region. Non-English literature and conference abstracts for which full-text articles were unavailable were excluded. Following screening and full-text evaluation, data were extracted and synthesized by topic. We also describes the clinical manifestations of *M. genitalium* infection and the molecular mechanisms underlying its resistance to macrolides, fluoroquinolones and tetracyclines. Furthermore, this paper maps global patterns of antimicrobial resistance, compares current treatment guidelines in the United States, Europe, East Asia, the United Kingdom and Australia, assesses the potential role of doxycycline post-exposure prophylaxis, and explores molecular epidemiological tools for genotyping and transmission network analysis. The main limitations of the available evidence, as well as the challenges facing clinical and public health management, are discussed. We aim to provide a basis for the development of evidence-based strategies to address the evolving threat of antimicrobial resistance in *M. genitalium*.

## 2 Clinical manifestations and drug resistance mechanisms of *M. genitalium* infection

### 2.1 Clinical manifestations

*M. genitalium*, as a significant sexually transmitted pathogen, can cause various genital tract diseases in both men and women (Gnanadurai and Fifer, 2020). In male patients, it is one of the main pathogens causing NGU (Horner and Martin, 2017), with typical symptoms including mucous or purulent secretions from the urethra, dysuria (such as frequent urination, urinary urgency, and painful urination), and a sensation of urethral itching (Bellinato et al., 2021). Infected women often present with cervicitis, which is characterized by cervical congestion, edema, and purulent discharge (Korte et al., 2006). Some patients may progress to PID, with lower abdominal pain, dyspareunia, and abnormal vaginal discharge (such as increased volume or abnormal odor) (Lis et al., 2015).

### 2.2 Acquired resistance mechanisms to three major classes of antibiotics

#### 2.2.1 Macrolide

**Figure 1:**
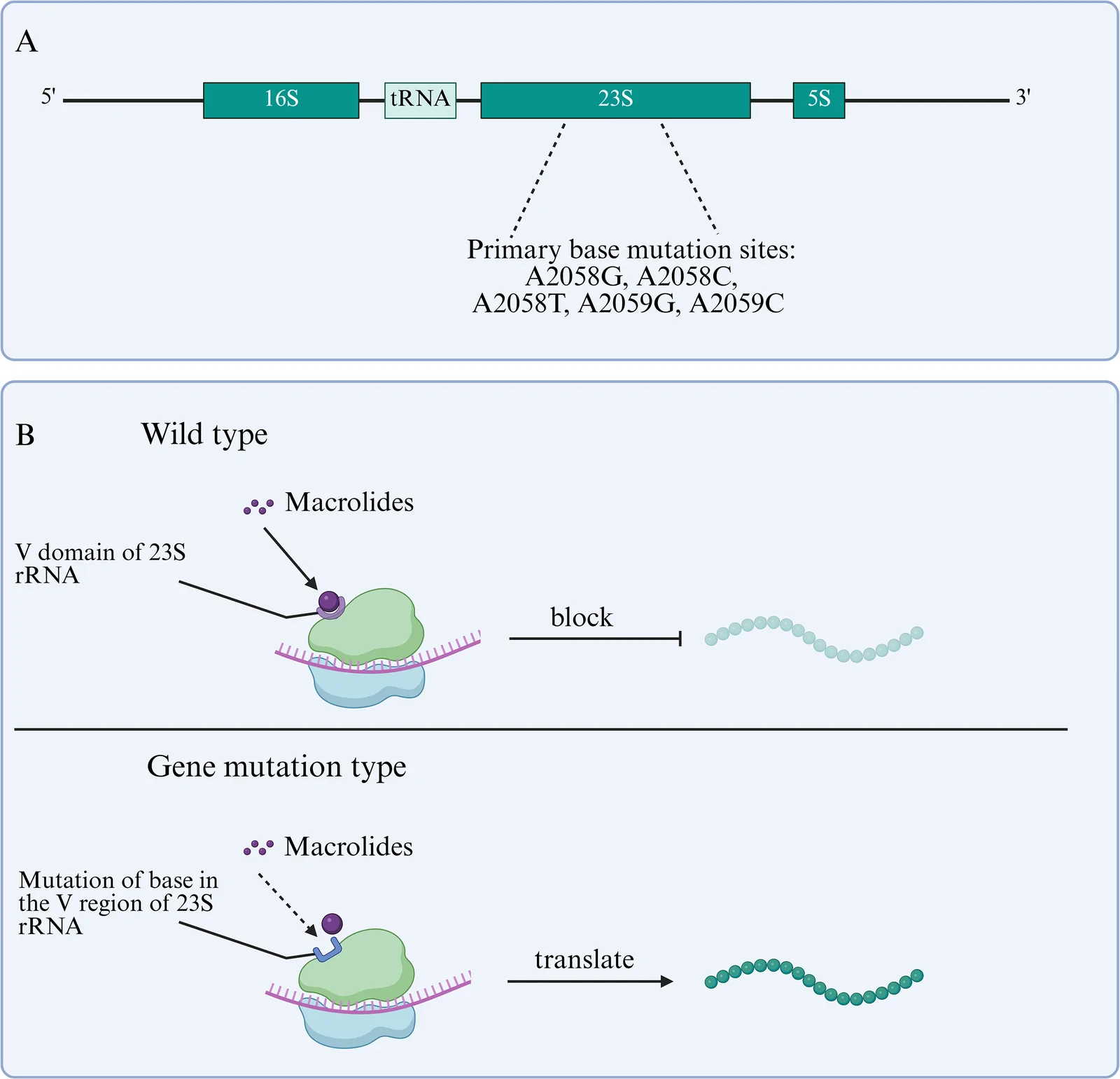
Mechanism diagram of macrolide antibiotic resistance. The mutation sites in bacterial rRNA responsible for macrolide resistance are primarily located within the 23S domain. The primary base mutation sites include A2058G, A2058C, A2058T, A2059G, A2059C, and others **(A)**. For wild type of *M. genitalium*, macrolides block protein synthesis by binding to the V domain of the bacterial 23S rRNA. For gene mutation type of *M. genitalium*, when single or multiple base mutations occur at specific sites on the 23S rRNA, the affinity between the drug and the ribosome decreases, leading to the development of drug resistance **(B)**.

23S rRNA gene mutation is the most common mechanism of drug resistance in *M. genitalium*. The major nucleotide mutation sites are A2058G, A2058C, A2058T, A2059G, and A2059C (Bissessor et al., 2015; Hamasuna et al., 2017; Harrison et al., 2019; Jin et al., 2020; Mahlangu et al., 2022; Azrad et al., 2024; Tsai et al., 2024) (**Figure 1A**). Macrolide antibiotics (such as azithromycin and erythromycin) bind to the V domain of bacterial 23S rRNA, thereby inhibiting protein synthesis. When single or multiple base mutations occur at one or more sites on the 23S rRNA, the affinity between the drug and the ribosome decreases, leading to the development of drug resistance (**Figure 1B**). Phenotypic antibiotic resistance refers to the method of culturing pathogens, either axenic or in co-culture with eukaryotic cells, and then adding antibiotics to the culture medium to determine the minimum inhibitory concentration (MIC). This method has now been successfully used to study the effects of mutations in the 23S rRNA and *parC* genes on the MIC (Hamasuna et al., 2005; Hamasuna et al., 2018). Phenotypic resistance of *M. genitalium* to macrolide antibiotics is primarily manifested as resistance to azithromycin; this resistance phenotype is mediated by a single-nucleotide mutation at position A2058 or A2059 of the 23S rRNA gene, which can significantly increase the MIC of azithromycin. It has also been shown that not all mutations in 23S rRNA has the same consequences regarding azithromycin MIC. Isolates carrying A2058T exhibited 24−fold, 7−fold, 15−fold and 12−fold stronger growth inhibition by azithromycin relative to isolates with A2058G at azithromycin concentrations of 4, 8, 16 and 32 mg/L, respectively (P < 0.01), which may account for the disparities observed in clinical treatment responses (Doelman et al., 2025). The prevalence of macrolide phenotypic resistance in *M. genitalium* has exceeded 50% in many regions worldwide; the widespread prevalence of this resistance phenotype has directly led to a significant increase in the failure rate of empirical treatment with macrolides (Jensen and Unemo, 2024).

Studies on *M. genitalium* resistance conducted in various regions of Asia have mentioned or confirmed resistance mechanisms associated with the 23S rRNA. Antimicrobial resistance surveillance in multiple regions of China has confirmed the presence of macrolide-resistance-associated mutations in the 23S rRNA gene of *M. genitalium*. For example, in a study conducted in Hong Kong, China, 42.1% of positive samples carried such mutations (Jin et al., 2020); a study in Taiwan, China showed that the mutations were primarily concentrated at the A2058 site (61.5%) and the A2059 site (38.5%) (**Table 1**) (Tsai et al., 2024); furthermore, a study of infertile individuals detected multiple mutation types, including A2059G (62.9%), A2058T (24.2%), A2058G (11.3%), and T2086C (1.6%) (Li et al., 2018). A study on the prevalence of antibiotic resistance in *M. genitalium* in Israel identified the A2059C mutation in 23SrRNA as the most common MRM (Azrad et al., 2024). In Japan, 23S rRNA V-region sequencing was performed on urine specimens collected from male urethritis patients between 2005 and 2016 to analyze mutations associated with macrolide antibiotic resistance. Between 2005–2009 and 2010-2016, 4.4% (4/90) and 40.3% (27/67) of Japanese male genomes, respectively, harbored mutations such as A2058G and A2059G associated with macrolide resistance (Hamasuna et al., 2017).

**Table 1:** Summary of acquired mechanisms of drug resistance.

Mechanisms of resistance	Molecular mechanisms	Molecular basis	Associated antibiotics	Reference(s)
Target site alteration	23S rRNA gene mutation	A2058G, A2058C, A2058T, A2059G, A2059C gene mutation	Macrolides	(Bissessor et al., 2015; Hamasuna et al., 2017; Harrison et al., 2019; Jin et al., 2020; Mahlangu et al., 2022; Azrad et al., 2024; Tsai et al., 2024)
	Amino acid mutations: ParC and ParE subunits (topoisomerase IV)	S83I,S83N, D87N gene mutation	Fluoroquinolones	(Yuan et al., 2024)
	Amino acid mutations: GyrA and GyrB subunits (DNA gyrase) These typically require synergistic action with ParC mutations to enhance resistance; isolated mutations result only in low-level resistance.	M95I, D99N gene mutation	Fluoroquinolones	(Tagg et al., 2013; Lewis and Couldwell, 2015)
Biofilm formation	Impedes drug penetration and enhances drug resistance	N/A	Various antibiotics	(Barik et al., 2022)
Ribosome protection	Protecting ribosomes from antibiotic damage	16S rRNA mutation	Tetracyclines	(Li et al., 2023)
Drug efflux	Protecting ribosomes from antibiotic damage	Major facilitator superfamily	Tetracyclines	(Paulsen et al., 1996)
Other mechanisms	May induce conformational changes in the ribosomal peptidyl transferase center, leading to reduced drug binding capacity.	N/A	Pleuromutilin	(Jana and Deb, 2006; Liu et al., 2020)

In addition, South Africa has conducted research on resistance mechanisms. Between 2015 and 2018, testing for 23S rRNA resistance‑associated mutations (RAMs) was performed on *M. genitalium*-positive specimens from the genital tract of adult male and female patients. The A2059G mutation in the 23S rRNA, associated with macrolide resistance, was detected (Mahlangu et al., 2022).

Several studies in Oceania have also explored the molecular mechanisms underlying *M. genitalium* drug resistance. A study conducted in Honiara, Solomon Islands, sequenced the V domain of the 23S rRNA region in *M. genitalium*. Positive samples underwent azithromycin resistance testing, with a particular focus on single nucleotide polymorphisms (SNPs) at positions 2058 and 2059 (Harrison et al., 2019). A study conducted in Queensland, Australia, from May to June 2016 aimed to determine the prevalence of *M. genitalium* and antibiotic resistance among international backpackers. It identified azithromycin RAMs in the 23S rRNA gene, including A2058G, A2058C, A2058T, A2059G, and A2059C (Trevis et al., 2018). Another study on drug resistance in *M. genitalium* patients from July 2012 to June 2013, conducted by Melanie Bissessor et al., found that MRMs were primarily A2059G/A2058G in the 23S rRNA gene (89%), followed by A2058C/A2059C (7%) and A2059T/A2058T (2%) (Bissessor et al., 2015).

A resistance mechanism related to 23S rRNA has also been demonstrated in European studies on drug resistance in *M. genitalium*. From 2010 to 2022, Russia and Belarus conducted a 12-year epidemiological study on the resistance of *M. genitalium* in pregnant women to macrolides and fluoroquinolones. To detect macrolide or fluoroquinolone resistance markers, real-time PCR with primer-mute probe fluorescence was employed. The results indicated that the most common mutation associated with macrolide resistance was A2059G (Avchinnikova et al., 2024). As can be seen, base mutations in the 23S rRNA of *M. genitalium* predominantly occur at positions 2058 and 2059, though other mutational regions may also contribute to the emergence of drug resistance.

#### 2.2.2 Fluoroquinolone

**Figure 2:**
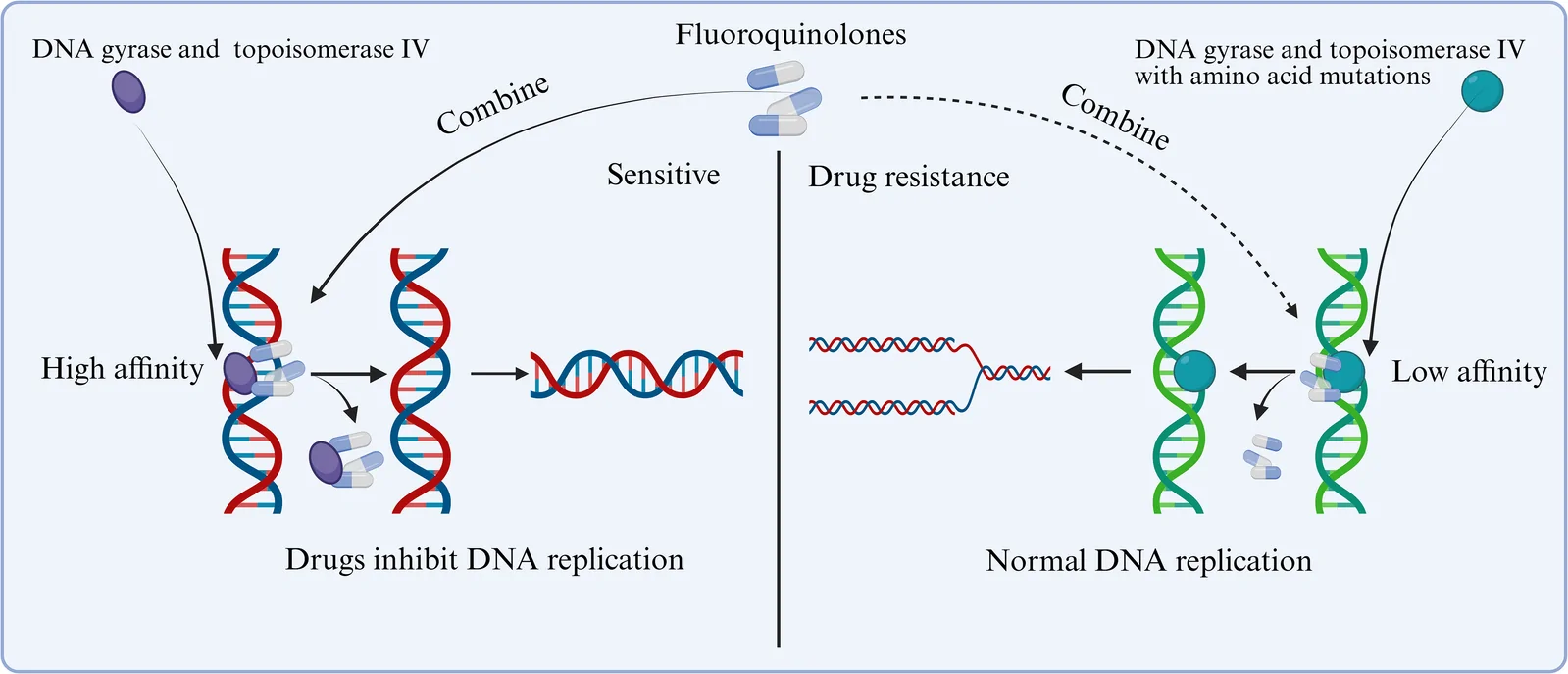
Mechanistic diagram of fluoroquinolone resistance due to mutations in DNA gyrase and topoisomerase IV genes. Fluoroquinolones exert their antibacterial effects by inhibiting the activity of DNA gyrase and topoisomerase IV, thereby affecting DNA function and structure. When mutations occur in the genes encoding these two enzymes, the drug’s ability to bind to topoisomerase IV is significantly reduced, allowing DNA to perform its normal functions.

Fluoroquinolones are key drugs for treating *M. genitalium* infections. The primary mechanism of resistance to fluoroquinolones in *M. genitalium* currently appears to be structural alterations in target enzymes caused by gene mutations. Multiple studies indicate that the resistance mechanism of *M. genitalium* to fluoroquinolones primarily involves mutations in the genes encoding DNA gyrase and topoisomerase IV. Fluoroquinolones exert their antibacterial effects by inhibiting DNA gyrase (composed of GyrA and GyrB subunits) and topoisomerase IV (composed of ParC and ParE subunits). Resistance in *M. genitalium* is primarily caused by mutations in the genes encoding these target enzymes, Mutations in the fluoroquinolone resistance determinant region of the *parC* gene are the primary cause. For instance, mutations at amino acid positions 81, 83, and 87 significantly reduce the drug’s binding affinity to topoisomerase IV (Tagg et al., 2013; Kikuchi et al., 2014; Lewis and Couldwell, 2015; Read et al., 2019; Vesty et al., 2020). Further studies have shown that phenotypic resistance of *M. genitalium* to fluoroquinolones is centered on resistance to moxifloxacin; this phenotype is primarily mediated by single-nucleotide polymorphisms at positions S83 and D87 in the quinolone resistance-determining region of the ParC gene and in the *gyrA* gene, which can significantly increase the MIC of moxifloxacin (Jensen and Unemo, 2024). In addition, the ParC S83I mutation in *M. genitalium* is the key molecular mechanism underlying moxifloxacin treatment failure. When mutations at the M95I and D99 sites of the *gyrA* gene coexist with the ParC S83I mutation, a significant synergistic effect occurs, nearly doubling the risk of moxifloxacin treatment failure compared to the ParC S83I mutation alone (Murray et al., 2023). Dual *parC/gyrA* gene mutations play a pivotal role in fluoroquinolone resistance. While *parC* mutations are the primary driver, concurrent *gyrA* mutations strongly enhance resistance levels through synergistic effects on target enzyme conformation. Such double mutations are closely associated with high-level resistance and poor clinical outcomes, underscoring their importance in resistance detection and therapeutic guidance (Ertl et al., 2023; Murray et al., 2023). Mutations in the fluoroquinolone resistance determinant region of the *gyrA* gene (such as methionine-to-isoleucine mutation at position 95, M95I) typically require synergistic action with *parC* mutations to enhance resistance; isolated mutations result only in low-level resistance (**Table 1**; **Figure 2**) (Tagg et al., 2013; Lewis and Couldwell, 2015). In terms of biofilm formation, some strains can form biofilms, potentially enhancing resistance by impeding drug penetration; yet, related studies are scarce and clinical significance remains unclear (Barik et al., 2022). The elucidation of these mechanisms provides crucial evidence for drug resistance testing and clinical treatment. For instance, guiding drug selection through detection of *parC* and *gyrA* mutations can enhance therapeutic efficacy and reduce the spread of resistance (Read et al., 2019; Vesty et al., 2020).

#### 2.2.3 Tetracycline

**Figure 3:**
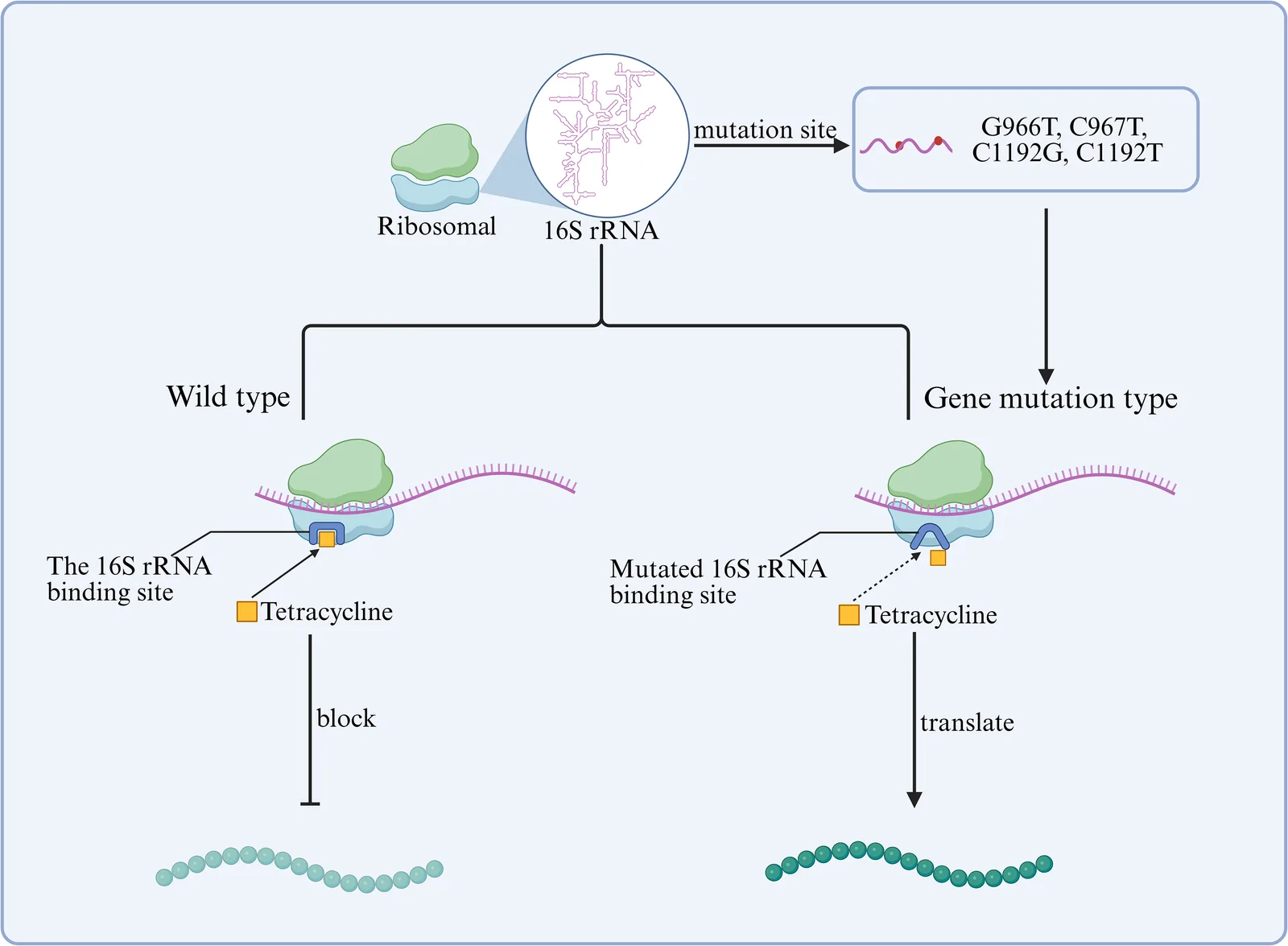
Mechanism of tetracycline resistance mediated by 16S rRNA mutations. The upper panel depicts the 16S rRNA structure within the 30S subunit of bacterial ribosomes. Base mutations associated with tetracycline resistance include: G966T, C967T, C1192G, C1192T, etc. The lower panel compares wild-type and mutant 16S rRNA: In wild-type *M. genitalium*, tetracycline antibiotics bind to 16S rRNA, inhibiting protein synthesis. However, when the 16S rRNA undergoes the aforementioned point mutations, the spatial conformation of the drug-binding site changes, preventing effective tetracycline binding and conferring resistance.

The mechanism of tetracycline resistance in *M. genitalium* primarily involves point mutations in the 16S rRNA gene. Tetracycline antibiotics inhibit protein synthesis by binding to the 16S rRNA of the 30S subunit of bacterial ribosomes. Mutations at specific sites within the 16S rRNA gene can alter the structure of the drug-binding site, thereby reducing drug affinity (**Figure 3**). Current studies have detected 16S rRNA mutations associated with tetracycline resistance in clinical specimens. For instance, the detection rate of the C1192T mutation in Chinese populations reached 22.5% (Li et al., 2023). It is worth noting that the mutation sites in the 16S rRNA gene are primarily concentrated in the H31 and H34 helical regions. However, the association between these two regions and tetracycline resistance has not yet been demonstrated in *M. genitalium* itself (Imai et al., 2026), and no clear relationship has been found between them and the MIC of tetracycline (Chua et al., 2022).

Beyond point mutations in the 16S rRNA gene, efflux pump mechanisms may also contribute to tetracycline resistance. Tetracycline efflux pumps, typically mediated by major facilitator superfamily (MFS) transporters, protect ribosomal function by reducing intracellular drug concentrations (Paulsen et al., 1996). In many bacteria, MFS-type tetracycline efflux pumps exhibit high specificity for tetracycline but limited activity against minocycline or glycylcycline. Efflux reduces intracellular drug concentrations, thereby protecting ribosomal function and mediating resistance.

#### 2.2.4 Others

Beyond the widely recognized mechanisms of resistance to macrolides, fluoroquinolones, and tetracyclines mentioned above, mechanisms of resistance to traditional drugs such as aminoglycosides and novel antimicrobial agents are also gradually gaining attention. Truncated side-chain peptide drugs (such as lefamulin) block translation initiation by binding to the peptidyl transferase center of bacterial 50S ribosomes, destabilizing tRNA and inhibiting the binding of Met-tRNA at the P site (Liao et al., 2021). This mechanism of action differs significantly from that of macrolides. No definitive resistance mechanisms have been identified to date. However, prolonged use may induce conformational changes in the ribosomal peptidyl transferase center, reducing drug binding affinity and potentially leading to resistance development. It is noteworthy that due to the distinct molecular mechanisms of action between this class of drugs and macrolides, the risk of cross-resistance between the two is relatively low. Relevant literature has conducted in-depth discussions on the mechanisms of action and resistance risks associated with Truncated side-chain peptide drugs (Jana and Deb, 2006). Oxazolidinone antibiotics (such as linezolid) act on bacterial ribosomes via a different target than macrolides, theoretically avoiding cross-resistance with macrolide antibiotics. However, long-term use still carries potential risks of resistance development. This may occur due to mutations in ribosome-related target genes (such as 23S rRNA or ribosomal proteins), leading to decreased drug binding affinity and subsequent resistance. To date, no such instances have been reported in *M. genitalium* (Liu et al., 2020). Relevant literature provides detailed explanations of its mechanism of action and related aspects of drug resistance (Machalek et al., 2020).

### 2.3 External drivers of resistance emergence and spread

#### 2.3.1 Antibiotic abuse and selective pressure

The misuse and inappropriate use of antibiotics constitute the primary external factors driving the emergence and evolution of *M. genitalium* drug resistance. Globally, the increasing incidence of STIs coupled with widespread antibiotic availability has led to the extensive use, even abuse, of antibiotics, particularly macrolides and fluoroquinolones. This has exerted potent selective pressure on *M. genitalium*, facilitating the selection and dissemination of drug-resistant strains (Machalek et al., 2020).

Azithromycin, as a representative macrolide antibiotic, has long been recommended in national guidelines as the first-line treatment for *M. genitalium* infections. However, the widespread clinical use of single-dose azithromycin regimens, particularly when administered empirically without microbiological diagnosis, has led to the rapid emergence and spread of resistant strains (Machalek et al., 2020). Research indicates that among patients who had used macrolide antibiotics within 30 days prior to their visit, the mutation rate of 23S rRNA (96.7%) was significantly higher than that observed in patients who had not used such drugs (86.8%) (Li et al., 2020b).

With the declining efficacy of azithromycin, fluoroquinolone antibiotics such as moxifloxacin have become second-line or rescue therapies for *M. genitalium* infections. However, inappropriate use of these drugs, including underdosing, incomplete treatment courses, or monotherapy, has similarly contributed to the development of resistance (Machalek et al., 2020).

#### 2.3.2 Sexually transmitted networks and the spread of drug-resistant strains

The characteristics and dynamic changes of sexual transmission networks play a crucial role in the spread of *M. genitalium* drug-resistant strains. These resistant strains can rapidly propagate through sexual contact networks, transforming drug resistance from an individual treatment failure into a population-level epidemiological issue. Sexual network structures involving multiple partners or concurrent partners facilitate the swift dissemination of drug-resistant strains. Within such sexual networks, once drug-resistant strains emerge, they can rapidly spread to multiple nodes (individuals) through sexual contact, leading to clustered transmission of these resistant strains (Li et al., 2020a).

#### 2.3.3 Asymptomatic transmission and drug-resistant spread

*M. genitalium* infection often presents as asymptomatic or with mild symptoms. Studies indicate that approximately 19.7% of infected individuals exhibit no corresponding clinical symptoms (Peel et al., 2020). These asymptomatic carriers often do not seek medical care or receive treatment, thereby becoming reservoirs and sources of transmission for drug-resistant strains. They unknowingly spread these resistant bacteria to new sexual partners. This hidden transmission pattern makes controlling the spread of drug resistance through traditional public health interventions exceptionally difficult.

#### 2.3.4 Limitations of diagnostic methods and the prevalence of empirical drug use

*M. genitalium* culture is time-consuming, making molecular diagnostic methods the widely adopted approach in clinical practice. However, even molecular diagnostics have limitations, particularly the low prevalence of drug resistance gene testing, which often leaves clinicians lacking precise evidence-based guidance for drug selection (Li et al., 2020b). Most clinical laboratories lack the capacity to routinely perform testing for *M. genitalium* resistance genes. Besides, associations between determinant detection and clinical treatment failure are not always clear. Furthermore, molecular assays have limited use for detection of emerging resistance mechanisms. If this sexually transmitted infection is to remain treatable, phenotypic susceptibility testing will play an invaluable role in evaluation of potential therapeutics (Pitt et al., 2022).

Physicians often select antibiotics based on experience, particularly in resource-limited areas where decisions are frequently guided by clinical symptoms and epidemiological data, lacking personalized antimicrobial susceptibility testing guidance (Li et al., 2020a). This empirical approach may lead to inappropriate antibiotic selection, insufficient treatment duration, or improper dosing, thereby accelerating the development of AMR. Furthermore, in cases where *M. genitalium* eradication has not been confirmed, the use of inappropriate treatment regimens may also lead to spontaneous mutations in residual bacterial strains (Bissessor et al., 2015). New diagnostic method such as ERA/CRISPR–Cas12a can be considered, as it has been proven to be a rapid, low-cost, ultrasensitive, and specific method for the diagnosis of *M. pneumoniae* (Deng et al., 2022).

## 3 Regional distribution of global drug resistance rates

**Figure 4:**
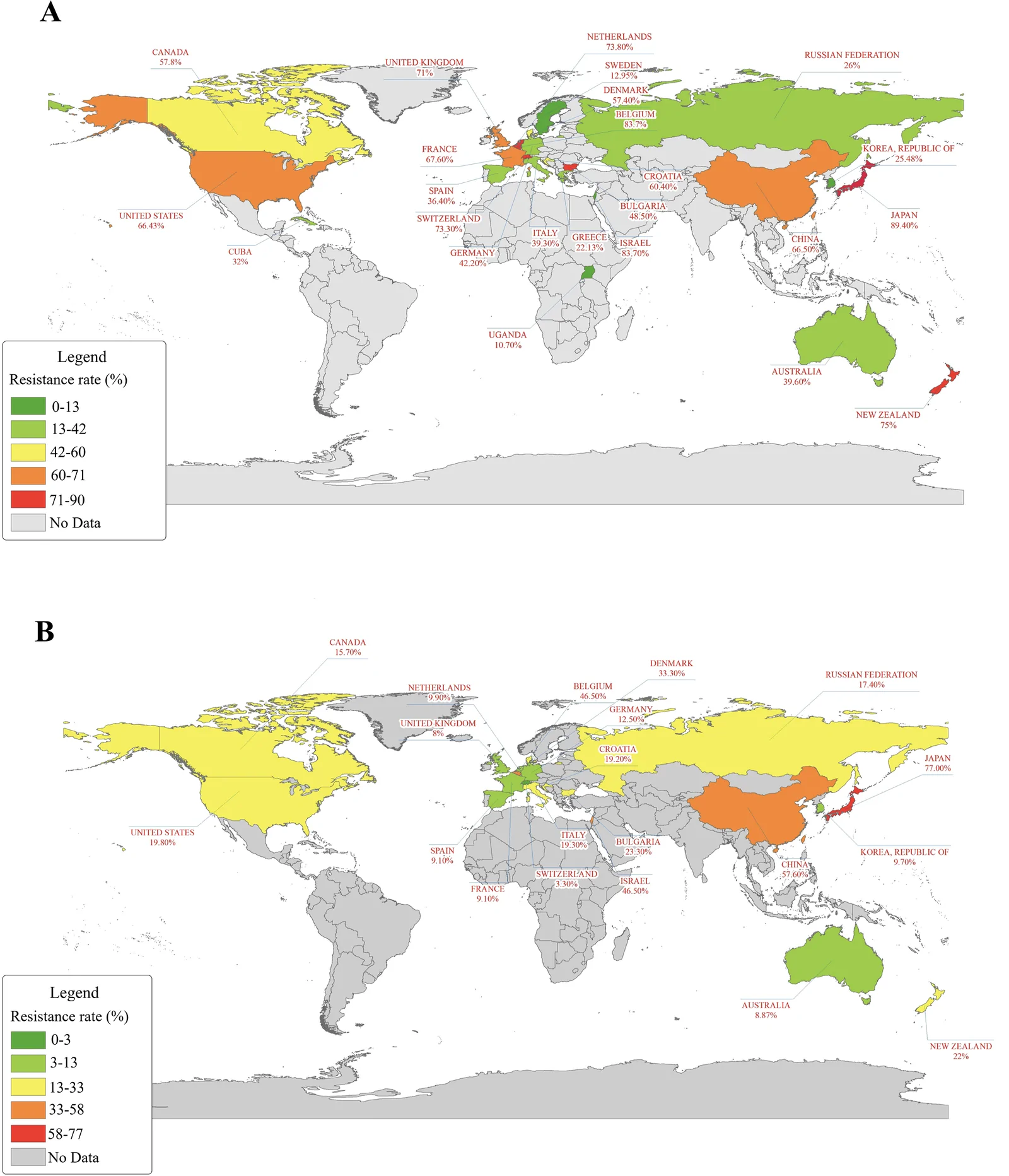
Global distribution of macrolide resistance rates **(A)** and fluoroquinolone resistance rates **(B)**. This map was generated using ArcGIS version 10.8. The resistance rates shown in the map are combined rates for the decade from 2015 to 2025, rather than cross-sectional data for a single year. The data are derived from published clinical studies, reviews, meta-analyses, etc. in PubMed, Embase, and MEDLINE databases. The combined drug resistance rate was calculated by relevant statistical methods (Freeman-Tukey double arcsine transformation, FTT). The color gradient from green to red represents the change of comprehensive resistance rate from low to high, and the gray area represents the country or region without available data. Jenks natural discontinuity classification method is used.

We compiled a global visualization of macrolide and fluoroquinolone resistance rates by region (**Figure 4**; **Tables 2**, **3**). There are obvious regional differences in the policy and practice of antibiotic use, which also contribute to the significant differences in the resistance rates of *M. genitalium* in different regions. For example, the prevalence of mutations associated with macrolide resistance was found to be significantly higher in samples from the World Health Organization (WHO) Western Pacific Region and the WHO Region of the Americas than in samples from the WHO European region (Machalek et al., 2020). This geographical variation may be related to population mobility patterns, the degree of sexual network connectivity, and antibiotic use policies in different regions. International human movement may also lead to the spread of drug-resistant strains across regions and accelerate the global spread of drug resistance.

**Table 2:** Global resistance to macrolide antibiotics.

Country	Region	Year of publication	Origin of samples	Number of samples screened	Identity of mutation	Frequency of mutation	Reference(s)
The Republic of South Africa	Johannesburg	2019	STI and HIV patients	266	23S rRNA	0%	(Muller et al., 2019)
	Multiple provinces	2022	Adult males and females presenting with genital discharge	182	23S rRNA	1.7%	(Mahlangu et al., 2022)
	Limpopo province	2022	Sexually active females	28	N/A	10.7%	(Melendez et al., 2022)
Japan	Tokyo	2023	MSM	141	23S rRNA	89.4%	(Ando et al., 2023b)
	Kyoto	2015	Female sex workers	149	23S rRNA	47.1%	(Deguchi et al., 2015)
South Korea	N/A	2021	The general population	208	A2059G	4.29%	(Seo et al., 2021)
Australia	Queensland	2017	N/A	67	N/A	63.6%	(Trembizki et al., 2017)
New Zealand	Auckland	2020	Community patients	48	23S rRNA	75%	(Vesty et al., 2020)
	Auckland	2020	Sexual health clinic patients	33	23S rRNA	76%	(Vesty et al., 2020)
Netherlands	The Southeastern Region	2019	The general population	124	N/A	21.8%	(Adelantado et al., 2019)
UK	N/A	2022	Asymptomatic patients, males with NGU, females with symptoms of pelvic inflammatory disease and patients requiring a test-of-cure in line with BASHH guidelines	389	A2058G, A2059G	71%	(Day et al., 2022)
	England	2021	Heterosexual males, females, MSM	249	A2058G, A2059G	69%	(Fifer et al., 2021)
Bulgaria	Sofia	2023	People with sexually transmitted infections	33	A2058G/T, A2059G	48.5%	(Philipova et al., 2023)
Germany	Berlin	2025	MSM	269	A2058G/T, A2059G	85.9%	(Dumke and Glaunsinger, 2025)
	Berlin	2025	Heterosexual patients	104	A2058G/T, A2059G	42.2%	(Dumke and Glaunsinger, 2025)
Russia	Moscow	2023	The general population	213	A2059G, A2058G	26%	(Edelstein et al., 2023)
Switzerland	Zurich	2022	MSM	30	N/A	86.7%	(Ring et al., 2022)
Italian	Bari	2021	The general population	47	A2058G	10.63%	(Angela et al., 2021)
Croatia	The Northwest Region	2024	Males	145	N/A	60.4%	(Ljubin-Sternak et al., 2024)
Netherlands	Amsterdam and Hague	2021	Heterosexual males, females, MSM	297	N/A	66.0%	(Hetem et al., 2021)
	N/A	2023	The general population	202	A2058G/T, A2059G/C	73.8%	(Yusuf et al., 2023)
Canada	Canada	2016	Female	963	23S rRNA	34.3%	(Chernesky et al., 2016)
	Toronto, Ontario	2016	The general population	50	A2058G, A2059G	58.0%	(Gesink et al., 2016)
	Montréal	2021	MSM	37	A2058G, A2059G	84.0%	(Labbé et al., 2021)
	Saskatchewan	2021	The general population	110	A2059G, A2059C, A2059T, A2058G, A2058C	63.6%	(Parmar et al., 2021)
Brazil	São Paulo	2025	MSM	33	23S rRNA	78.8%	(Passarelli et al., 2025)
	São Paulo	2025	MSM	26	A2058G	53.9%	(Passarelli et al., 2025)
	São Paulo	2025	MSM	26	A2058T	26.9%	(Passarelli et al., 2025)
	São Paulo	2025	MSM	26	A2059G	19.2%	(Passarelli et al., 2025)
Cuba	Cuba	2018	The general population	280	A2058G, A2059G, A2058C, A2058T	32.0%	(Mondeja et al., 2018)
China	Nanjing	2024	The general population	19	A2058T, A2058G, A2059G, A2059C	100%	(Yuan et al., 2024)
	Guangzhou	2023	The general population	89	A2058C, A2058G, A2058T, A2059C, A2059G, A2059T	64.0%	(Li et al., 2023)
	Shenzhen	2024	The general population	180	A2071G, A2071T, A2072G, A2075T	83.9%	(Wang et al., 2024)
	Hong Kong	2020	Female	19	A2071G, A2072G	42.1%	(Ng et al., 2020)
	Taiwan	2024	The general population	107	A2058, A2059	24.3%	(Tsai et al., 2024)
United States	Los Angeles	d2018	Clinical remnants	10	A2058G, A2059G	80.0%	(Allan-Blitz et al., 2018)
	Southwest	2021	Pregnant females	26	A2058G, A2059G, A2058T	30.8%	(Stafford et al., 2021)
	Alabama	2018	HIV-positive MSM	27	A2058G, A2059G	74.1%	(Dionne-Odom et al., 2018)
	Alabama	2020	African American couples	28	A2058G, A2059G	60.7%	(Xiao et al., 2019)
	Seattle	2020	Females with STD (vaginal)	9	A2058G, A2059G	89.0%	(Khosropour et al., 2020)
	Seattle	2020	Females with STD (rectal)	8	A2058G, A2059G	100%	(Khosropour et al., 2020)
	Los Angeles	2024	Males with urethritis	11	23S rRNA	81.8%	(Sukhija-Cohen et al., 2024)
	Baltimore	2021	Pregnant females	51	23S rRNA	N/A	(Perin et al., 2021)
France	N/A	2021	MSM	210	23S rRNA	67.6%	(Berçot et al., 2021)
	N/A	2016	The general population	106	23S rRNA	5.7%	(Le Roy et al., 2016)
	Montpellier	2021	MSM	96	23S rRNA	58.3%	(Guiraud et al., 2021)
	N/A	2023	The general population	1630	23S rRNA	34.7%~42.9%	(Pereyre et al., 2023)
Spain	Barcelona	2020	The general population	83	N/A	36.1%	(Fernández-Huerta et al., 2020)
	Barcelona	2017	N/A	N/A	N/A	35%	(Barberá et al., 2017)
	Lleida	2024	N/A	N/A	A2059G point mutation in 23S rRNA	57.9%	(Fraile García et al., 2024)
	Southern region	2021	24 MSM, 30 male patients, and 23 female patients	77	N/A	36.4%	(de Salazar et al., 2021)
	Santiago	2021	The general population	28	A2059G, A2058G, A2058T point mutation in 23S rRNA	20%	(Treviño et al., 2021)
Sweden	Stockholm	2017	The general population	171	N/A	18%	(Björnelius et al., 2017)
	Scania	2025	The general population	55	N/A	20%	(Lindroth et al., 2025)
	Scania	2017	The general population	239	N/A	13%	(Forslund et al., 2017)

STI, sexually transmitted infection;MSM, men who have sex with men; NGU, non-gonococcal urethritis; BASHH, British Association of Sexual Health and HIV; STD, sexually transmitted diseases.

**Table 3:** Global resistance to fluoroquinolone antibiotics.

Country	Region	Year of publication	Origin of samples	Number of samples screened	Identity of mutation	Frequency of mutation	Reference(s)
The Republic of South Africa	Johannesburg	2019	STI and HIV patients	266	*gyrA* and *parC*	0.4%	(Muller et al., 2019)
Japan	Tokyo	2023	MSM	120	*gyrA*	22.5%	(Ando et al., 2023b)
	Kyoto	2015	Female sex workers	149	*parC*	36.8%	(Deguchi et al., 2015)
	Kyoto	2018	Male patients with acute urethritis	58	*parC*, *gyrA* and 23S rRNA triple mutations	7.7%	(Deguchi et al., 2018)
	Kyoto	2021	MSM	36	*parC* and *gyrA* dual mutations	25.0%	(Ando et al., 2021)
	Osaka	2018	Male patients with acute urethritis	32	*parC*, *gyrA* and 23S rRNA triple mutations	19.2	(Deguchi et al., 2018)
	Sendai	2018	Male patients with acute urethritis	23	*parC*, *gyrA* and 23S rRNA triple mutations	8.7%	(Deguchi et al., 2018)
South Korea	N/A	2021	The general population	208	*parC*	12.86%	(Seo et al., 2021)
New Zealand	Auckland	2020	Community patients	48	*parC* and *gyrA*	19%	(Vesty et al., 2020)
	Auckland	2020	Sexual health clinic patients	33	*parC* and *gyrA*	27%	(Vesty et al., 2020)
UK	N/A	2022	Asymptomatic patients, males with NGU, females with symptoms of pelvic inflammatory disease and patients requiring a test-of-cure in line with BASHH guidelines	389	S83I/R, D87Y/N	8%	(Day et al., 2022)
	England	2021	Heterosexual males, females, MSM	251	S83I/R, D87Y/N	8%	(Fifer et al., 2021)
Bulgaria	Sofia	2023	People with sexually transmitted infections	68	S83I, D87N, S84P	23.3%	(Philipova et al., 2023)
Germany	Berlin	2025	MSM	269	S83I, D87Y/N/G,W100R, S83N	19.7%	(Dumke and Glaunsinger, 2025)
	Berlin	2025	Heterosexual patients	104	S83I, D87Y/N, S83N	12.5%	(Dumke and Glaunsinger, 2025)
Russia	Moscow	2023	The general population	213	S80I/N, D84Y/N/G,	17%	(Edelstein et al., 2023)
Switzerland	Zurich	2022	MSM	30	N/A	16.7%	(Ring et al., 2022)
Croatia	the Northwest Region	2024	Males	145	N/A	19.2%	(Ljubin-Sternak et al., 2024)
Netherlands	N/A	2023	The general population	202	G248A/T, G259A/C/T, A247C	9.9%	(Yusuf et al., 2023)
Canada	Canada	2016	The general population	110	*gyrA* and *parC*	22.4%	(Chernesky et al., 2016)
	Toronto, Ontario	2016	The general population	50	G248T, G259A (*parC*)	20.0%	(Gesink et al., 2016)
	Montréal	2021	MSM	37	G248T, G248A, A247C(*parC*)	32.0%	(Labbé et al., 2021)
	Saskatchewan	2021	The general population	85	S83I(*parC*)	10.6%	(Parmar et al., 2021)
Brazil	São Paulo	2025	MSM	38	*parC*	21.1%	(Passarelli et al., 2025)
	São Paulo	2025	MSM	8	G248T (*parC*)	75.0%	(Passarelli et al., 2025)
	São Paulo	2025	MSM	8	A247C (*parC*)	25.0%	(Passarelli et al., 2025)
China	Nanjing	2024	The general population	18	G248T (*parC*)	61.1%	(Yuan et al., 2024)
	Nanjing	2024	The general population	15	G277T (*gyrA*)	6.7%	(Yuan et al., 2024)
	Nanjing	2024	The general population	14	C1384T (gyrB)	7.1%	(Yuan et al., 2024)
	Guangzhou	2023	The general population	77	Ser83Ile, Ser83Asn, Ser83Arg, Asp87Gly, Asp87Asn, Asp87Tyr (*parC*)	67.5%	(Li et al., 2023)
	Guangzhou	2023	The general population	160	Ser83Ile, Ser83Asn, Asp87Asn, Asp87Tyr, Gly81Cys(*parC*)	73.8%	(Li et al., 2023)
	Shenzhen	2024	The general population	163	Met95Ile, Asp99Tyr, Asp107Asn(*gyrA*)	2.5%	(Wang et al., 2024)
	Hong Kong	2020	Female	20	G248T, G259T, G259A(*parC*)	42.1%	(Ng et al., 2020)
	Taiwan	2024	The general population	116	Gly81, Ser83, Asp87(*parC*)	22.4%	(Tsai et al., 2024)
The United	Southwest	2021	Pregnant females	18	*parC* (S83N)	5.6%	(Stafford et al., 2021)
	Southwest	2021	Pregnant females	26	*gyrA*	3.8%	(Stafford et al., 2021)
	Alabama	2018	HIV-positive MSM	27	*parC* (S83I/S83R)	29.6%	(Dionne-Odom et al., 2018)
	Alabama	2020	African American couples	28	*parC* (S83I)	11.1%	(Xiao et al., 2019)
	Seattle	2020	Females with STD	16	*parC*	0%	(Khosropour et al., 2020)
	Los Angeles	2024	Males with urethritis	11	*parC*	9.1%	(Sukhija-Cohen et al., 2024)
	Los Angeles	2018	Clinical remnants	10	Not tested	N/A	(Allan-Blitz et al., 2018)
	Baltimore	2021	Pregnant females	51	Not tested	N/A	(Perin et al., 2021)
France	N/A	2021	MSM	210	Topoisomerase IV gene mutation	9.1%	(Berçot et al., 2021)
	Montpellier	2021	MSM	95	N/A	10.8%	(Guiraud et al., 2021)
	N/A	2023	The general population	1630	N/A	14.9%~16.1%	(Pereyre et al., 2023)
Spain	Barcelona	2017	N/A	N/A	N/A	8%	(Barberá et al., 2017)
	Southern region	2021	24 MSM, 30 male patients, and 23 female patients	77	N/A	9.1%	(de Salazar et al., 2021)

See abbreviations in **Table 2**.

## 4 Guidelines for *M. genitalium* infections treatment and emerging prevention and control issues

### 4.1 Comparative analysis of mainstream treatment guidelines

Based on local clinical practices, different regions have developed differentiated treatment recommendations. To facilitate a direct comparison of the key points of these guidelines, they are summarized in **Table 4**. As supported by current medical literature and regional epidemiological data, the clinical management of *M. genitalium* infections varies significantly among the United States, Europe, Japan, South Korea, the United Kingdom and Australia. These differences primarily stem from regional variations in antibiotic resistance patterns, drug availability, and antimicrobial stewardship philosophies. The United States follows the CDC guidelines, which employ a standardized sequential therapy strategy, administering doxycycline to all patients initially to reduce pathogen load, followed by stratified use of azithromycin or moxifloxacin based on resistance status, while strictly prohibiting single-dose azithromycin to prevent resistance (Workowski et al., 2021). Europe, guided by EADV and WHO recommendations, promotes precision medicine based on molecular diagnostics, determining the treatment path according to 23S rRNA gene mutation testing, using a doxycycline-azithromycin combination for sensitive genotypes, and directly escalating to moxifloxacin for resistant genotypes, with pristinamycin reserved as a salvage therapy (Jensen et al., 2022). Japan adopts an aggressive eradication strategy with sequential doxycycline and azithromycin, a 14-day moxifloxacin regimen for complicated infections such as PID, and ongoing assessment of sitafloxacin as a promising second-line option (Ando et al., 2023a). South Korea recommends a total 1.5 g multi-day azithromycin regimen for initial treatment and adopts a two-stage therapeutic principle, applying doxycycline for pathogen reduction before stratified adjustment and moxifloxacin escalation for suspected resistant strains (Lee et al., 2024). The UK implements resistance-tailored sequential regimens recommended by BASHH (British Association of Sexual Health and HIV), using doxycycline followed by azithromycin for macrolide-susceptible strains and moxifloxacin for resistant cases (Soni et al., 2025), while Australia formulates its therapeutic protocols based on Australasian Society for HIV Medicine (ASHM) guidelines with standardized sequential strategies and routine post-treatment cure evaluation for *M. genitalium* infections (Adawiyah et al., 2024).

**Table 4:** International clinical guidelines for the treatment of *M. genitalium* infection.

Region	Treatment strategy	First-line therapy	Second-line and salvage therapy	Reference(s)
Europe	Resistance-guided sequential therapy	Doxycycline followed by azithromycin.	Moxifloxacin. Salvage therapy: pristinamycin.	(Jensen et al., 2022)
United States	Empirical sequential treatment strategy	Doxycycline followed by azithromycin.	Doxycycline followed by moxifloxacin. Alternative: minocycline.	(Workowski et al., 2021)
Japan	Empirical monotherapy or sequential therapy	Azithromycin single-dose or Doxycycline.	Sitafloxacin. Salvage therapy: sequential Doxycycline combined with sitafloxacin.	(Ando et al., 2023a)
South Korea	Stratified treatment based on antimicrobial susceptibility	Azithromycin.	Doxycycline followed by high-dose azithromycin or moxifloxacin.	(Lee et al., 2024)
United Kingdom	Mandatory pre-treatment macrolide resistance testing and sequential therapy	Doxycycline followed by azithromycin.	Doxycycline followed by moxifloxacin. Salvage therapy: pristinamycin.	(Soni et al., 2025)
Australia	Resistance-testing prioritized strategy	Doxycycline followed by azithromycin.	Doxycycline followed by moxifloxacin Salvage therapy: pristinamycin.	(Adawiyah et al., 2024)

### 4.2 Potential effects of doxycycline post-exposure prophylaxis

DoxyPEP, consisting of a single 200 mg dose of doxycycline taken within 72 hours post-unprotected intercourse, is a highly promising intervention (Fernandez-Ibanez et al., 2025). When DoxyPEP is administered for post-exposure prevention, its core mechanism lies in interfering with early pathogen replication, which inhibits the establishment of infection. Therefore, although doxycycline alone is not effective as a treatment regimen for curing confirmed *M. genitalium* infection (with a cure rate of only about 30%-40% for monotherapy), it can play a positive role in a post-exposure prophylaxis strategy (reducing pathogen load and the risk of macrolide resistance selection) (Jensen et al., 2022). While the current severe resistance challenges faced by *M. genitalium* mainly focus on macrolides and fluoroquinolones, rather than tetracyclines such as doxycycline (Wen et al., 2023; Chua et al., 2025b), *M. genitalium* exhibits relatively low natural susceptibility to doxycycline (i.e., low intrinsic sensitivity), and current studies have not found clear evidence of acquired tetracycline resistance (Fernandez-Ibanez et al., 2025). However, since DoxyPEP itself is a core component of combination therapy regimens for treating *M. genitalium* infection (as indicated in all articles in section 4.1 that include DoxyPEP as a core treatment) (Jensen et al., 2022; Lee et al., 2024; Soni et al., 2025) with the promotion of DoxyPEP as a preventive strategy in high-risk populations, strengthening molecular epidemiological surveillance of tetracycline resistance in *M. genitalium* becomes particularly important (Fernandez-Ibanez et al., 2025; Jenks and Semchenko, 2025). Additionally, doxycycline affects the composition of different microbial communities. Therefore, Europe currently does not recommend the use of DoxyPEP and further research is urgently needed to clarify the specific patient population characteristics that meet the criteria for this treatment strategy (Fernandez-Ibanez et al., 2025) (Bébéar et al., 2023). At present, DoxyPEP offers a promising way to reduce the infectious burden of this stubborn pathogen, *M. genitalium*, but its potential risks cannot be ignored.

### 4.3 Molecular epidemiological methods for identifying different genotypes and transmission chains

Examining the transmission dynamics and genetic evolution of *M. genitalium* from a molecular epidemiological perspective, and combining high-resolution molecular genotyping techniques with resistance data, is key to revealing the patterns of resistance transmission and is indispensable for a comprehensive understanding of the regional distribution of resistance. Currently, molecular typing methods with sufficient discriminatory power, stability, and reproducibility—such as the analysis of genes containing repetitive sequences, the analysis of variable genomic regions without repetitive sequences, the determination of single-locus and multi-locus variable-number tandem repeats (VNTRs), and the detection of SNPs in different genes—have been progressively adopted (Dumke, 2022). For example, Piñeiro et al. amplified and sequenced the MG191 adhesin, MG309 lipoprotein, and rRNA operon, and performed genotyping using phylogenetic analysis, VNTR analysis, and SNP analysis. This genotyping method can distinguish between persistent and recurrent infections, indicating that mutations associated with macrolide resistance primarily occur during treatment (Piñeiro et al., 2019). Adriaens et al. analyzed the epidemiological characteristics of *M. genitalium* in isolates from men diagnosed with NGU based on *mgpB/MG309* molecular genotyping and compared men who have sex with men (MSM) and MSW populations, revealing two groups that differed in sexual orientation and antibiotic resistance, highlighting the importance of monitoring resistance in different genotypes (Adriaens et al., 2025; Chen et al., 2025b). Furthermore, Ma et al. identified new potential genetic markers through short tandem repeat (STR) analysis of published *M. genitalium* genome sequences and applied both known and newly discovered markers to a set of clinical isolates to determine the optimal combination for a high-throughput multiplex genotyping system. The study found that the combination of *MG309*-STR and *MG191*-SNP is suitable for general epidemiological studies, while the addition of MG307-STR and *MG338*-STR may aid in studies of the sexual transmission networks of *M. genitalium* infections (Ma et al., 2008; Qiu et al., 2024). Chua et al. used whole-genome sequencing (WGS) to identify a novel 23S rRNA gene mutation in *M. genitalium*, expanding our understanding of resistance mechanisms within the genus *Mollicutes* (Yu et al., 2023; Chua et al., 2025a). By integrating these molecular epidemiological data with information on drug resistance phenotypes and genotypes, it is possible to elucidate the microevolutionary mechanisms underlying differences in drug resistance profiles across regions, thereby providing an evidence-based foundation for developing targeted interventions to halt the regional spread of drug-resistant strains. Therefore, strengthening the application of molecular epidemiological methods in future research will help curb the further spread of *M. genitalium* drug resistance at its source.

## 5 Limitations and challenges

Regarding the emergence and evolution of drug resistance, AMR can develop through the selective pressure of antibiotic exposure (inducing mutations during treatment or selecting for pre-existing mutations). Resistance to both first-line and second-line treatment regimens is increasing globally (Mahlangu et al., 2022), with limited alternative treatment options available, raising significant concerns (Gnanadurai and Fifer, 2020; Dai et al., 2021). Limitations of detection methods: Commonly used genotypic testing (detecting drug-resistant mutations) has shortcomings, such as unclear associations with clinical treatment failure, limited detection capabilities for emerging resistance mechanisms, and restricted applicability when the significance of certain mutations remains unclear (He et al., 2023). Challenges in treatment and management include inconsistent global therapeutic strategies and the absence of a clearly defined optimal regimen. Single-dose macrolides have been removed from first-line treatment guidelines in multiple countries. Recommendations for cure testing remain controversial, as excessive testing and treatment may contribute to the emergence of multidrug-resistant strains. Indiscriminate screening of asymptomatic individuals increases antimicrobial consumption and resistance development, necessitating strict adherence to testing guidelines (Pitt et al., 2022).

In terms of diagnostic and research challenges, the difficulty in the lack of widely available diagnostic methods (such as routine PCR) has led to the widespread use of empirical treatment, exacerbating drug resistance (Deng et al., 2018; Sweeney et al., 2022). Phenotypic antimicrobial susceptibility testing is only performed in a limited number of specialized laboratories, making it difficult to scale up. Furthermore, its refinement and standardization face complex challenges and significant economic burdens, with low-income countries facing particularly severe constraints. Insufficient knowledge and surveillance regarding the transmission, life cycle, pathogenesis, and spread of drug resistance among clonal/strain lineages of *M. genitalium* necessitate enhanced public health monitoring and global information dissemination to refine treatment strategies (Htaik et al., 2025). Regarding regional and population variations, significant differences in antibiotic resistance rates exist across different regions and populations. For instance, French studies indicate that men exhibit significantly higher macrolide resistance rates (52.4%-60.2%) compared to women (15.9%-22.2%). In Belgian male samples, 72.4% showed macrolide resistance, while all male-male sexual contact MSM samples carried relevant resistance mutations. Such disparities complicate the development of uniform prevention and control strategies (Ljubin-Sternak et al., 2024). Regarding the complexity of dynamic changes in drug resistance, retesting patients reveals that resistance profiles evolve over time. It remains unclear whether changes in resistance observed in repeated samples stem from alterations in the same strain or new infections by different strains. This ambiguity complicates tracking the trajectory of resistance development and assessing the impact of treatment on resistance, posing significant challenges for adjusting clinical treatment regimens. Regarding limitations in detection scope, current testing for fluoroquinolone resistance primarily focuses on the *parC* gene, overlooking the potential impact of mutations in the *gyrA* gene. Although *gyrA* mutations are less common and often coexist with *parC* mutations, the combined effect of non-synonymous mutations in both genes can exacerbate resistance. The current insufficient detection scope may underestimate actual resistance levels, thereby compromising the effectiveness of treatment strategies (Luo et al., 2023). Research limitations include inadequate sample representativeness (e.g., exclusively male participants from specific regions), selection bias (predominantly symptomatic patients seeking medical care), and insufficient clinical epidemiological and sexual behavior data. Furthermore, difficulties in obtaining detailed treatment information and follow-up efficacy data restrict the generalizability of findings and hinder comprehensive analysis of factors influencing drug resistance. This impedes deeper understanding of resistance mechanisms and optimization of prevention and control strategies (Munson et al., 2023; Yu et al., 2023). Additionally, vaccine development represents an effective strategy to prevent *M. genitalium* infection and curb the spread of drug-resistant strains (Qin et al., 2019; Rehman et al., 2025), as a vaccine against *M. pneumoniae* has been shown to have a protective effect in animals (Jiang et al., 2021; Zuo et al., 2024; Zeng et al., 2025).

## 6 Conclusion

This review provides an overview of the mechanisms and regional distribution of drug resistance in *M. genitalium*. It summarizes the acquired resistance of *M. genitalium* to current therapeutic agents and its external drivers, compiles recent global data on resistance distribution, and examines national treatment guidelines and emerging issues. However, significant limitations, such as the dynamic nature of resistance, limited diagnostic access, few alternative treatments, and inconsistent therapeutic strategies, severely hamper both research and the management of resistant infections. To address these challenges, future research should focus on generating higher-quality epidemiological data, strengthening molecular epidemiological methods, and deepening the understanding of resistance mechanisms. Accelerating the development of vaccines and novel therapeutics holds great promise for infection prevention and resistance containment. Future prevention and control efforts should therefore prioritize these innovations and the formulation of feasible strategies tailored to resource-limited settings.
